# Assessing the acceptability and feasibility of reactive drug administration for malaria elimination in a *Plasmodium vivax* predominant setting: a qualitative study in two provinces in Thailand

**DOI:** 10.1186/s12889-023-15852-z

**Published:** 2023-07-13

**Authors:** Kanokwan Suwannarong, Chris Cotter, Thanomsin Ponlap, Nisachon Bubpa, Kannika Thammasutti, Jintana Chaiwan, Timothy P. Finn, Suravadee Kitchakarn, Andreas Mårtensson, Kimberly A. Baltzell, Michelle S. Hsiang, Cheewanan Lertpiriyasuwat, Prayuth Sudathip, Adam Bennett

**Affiliations:** 1 Center of Excellence for Emerging and Re-Emerging Infectious Diseases in Animals, Faculty of Veterinary Science, Chulalongkorn University, Bangkok, Thailand; 2 SUPA71 Co., Ltd, Bangkok, Thailand; 3 Malaria Elimination Initiative, Institute for Global Health Sciences, University of California San Francisco, 550 16th Street, 3rd floor, San Francisco, CA 94158 USA; 4 Department of Women’s and Children’s Health, Uppsala University, Uppsala, Sweden; 5 Faculty of Nursing, Khon Kaen University, Khon Kaen Province, Thailand; 6 Department of Disease Control, Division of Vector Borne Diseases, Ministry of Public Health, Nonthaburi, Thailand; 7 Institute for Global Health Sciences, University of California San Francisco, San Francisco, CA USA; 8 School of Nursing, University of California San Francisco, San Francisco, CA USA; 9 Department of Epidemiology and Biostatistics, University of California San Francisco, San Francisco, CA USA; 10 Department of Pediatrics, University of California San Francisco, Benioff Children’s Hospital, San Francisco, CA USA; 11 PATH, Seattle, WA USA

**Keywords:** Reactive drug administration, Feasibility, Acceptability, Malaria elimination, Thailand

## Abstract

**Background:**

Reactive case detection (RACD) or testing and treatment of close contacts of recent malaria cases, is commonly practiced in settings approaching malaria elimination, but standard diagnostics have limited sensitivity to detect low level infections. Reactive drug administration (RDA), or presumptive treatment without testing, is an alternative approach, but better understanding regarding community acceptability and operational feasibility are needed.

**Methods:**

A qualitative study was conducted as part of a two-arm cluster randomized-controlled trial evaluating the effectiveness of RDA targeting high-risk villages and forest workers for reducing *Plasmodium vivax* and *P. falciparum* malaria in Thailand. Key informant interviews (KIIs) and focus group discussions (FGDs) were conducted virtually among key public health staff, village health volunteers (VHVs), and household members that implemented or received RDA activities. Transcriptions were reviewed, coded, and managed manually using Dedoose qualitative data analysis software, then underwent qualitative content analysis to identify key themes.

**Results:**

RDA was well accepted by household members and public health staff that implemented it. RDA participation was driven by fear of contracting malaria, eagerness to receive protection provided by malaria medicines, and the increased access to health care. Concerns were raised about the safety of taking malaria medicines without having an illness, particularly if underlying health conditions existed. Health promotion hospital (HPH) staff implementing RDA noted its operational feasibility, but highlighted difficulty in traveling to remote areas, and requested additional travel resources and hiring more VHVs. Other challenges were highlighted including the need for additional training for VHVs on malaria activities and the inability of HPH staff to conduct RDA due to other health priorities (e.g., Covid-19). More training and practice for VHVs were noted as ways to improve implementation of RDA.

**Conclusions:**

To maximize uptake of RDA, regular education and sensitization campaigns in collaboration with village leaders on the purpose and rationale of RDA will be critical. To alleviate safety concerns and increase participant safety, a rigorous pharmacovigilance program will be important. To accelerate uptake of RDA, trust between HPH staff and VHVs and the communities they serve must continue to be strengthened to ensure acceptance of the intervention.

**Trial registration:**

This study was approved by the Committee on Human Research at the University of California San Francisco (19–28,060) and the local Ethics Committee for Research in Human Subjects at Tak Provincial Health office (009/63) and Kanchanaburi Provincial health office (Kor Chor 0032.002/2185). Local authorities and health officers in the provinces, districts, and villages agreed upon and coordinated the implementation of the study. All methods in this study were carried out in accordance with relevant guidelines and regulations.

**Supplementary Information:**

The online version contains supplementary material available at 10.1186/s12889-023-15852-z.

## Background

Reactive case detection (RACD) is a widely-practiced malaria elimination strategy whereby household and nearby community members of a passively-identified index patient are tested with either a rapid diagnostic test (RDT) or by microscopy [1, 2]. However, standard diagnostics have limitations to detect low-level and *Plasmodium vivax* infections, which is commonly the dominant parasite species in elimination settings [3]. *P. vivax* has a dormant liver stage where the malaria parasites can evade detection, [4]. Also, lower levels of parasitemia may be able to transmit malaria [5].

Reactive drug administration (RDA), the presumptive treatment of household and nearby community members of a passively-detected index patient, is a potential approach to overcome the issue of low diagnostic sensitivity, and may be an effective strategy at interrupting transmission particularly in areas with persistent malaria foci. Recent studies in *P. falciparum*-dominant settings have shown the effectiveness of RDA in reducing malaria transmission with high study population coverage [6] and safety of RDA [7]. We recently conducted a two-arm cluster randomized-controlled trial (RCT) of RDA in a *P. vivax* predominant setting in Thailand [8]. The results regarding the effectiveness for reducing transmission will be reported elsewhere. Here, we report the acceptability and feasibility of the intervention, factors that are critical to ensuring high population coverage [9, 10].

Targeted mass antimalarial drug administration pilots in the Greater Mekong Subregion highlight the importance of understanding target communities to provide appropriate information in suitable ways [11]. Some evidence exists on the acceptability and operational feasibility of RDA in a low transmission *P. falciparum* settings, [6, 7, 12, 13]. Although these findings have relevance in all settings, *P. vivax* predominant settings have unique challenges due to the treatment. Specifically, radical cure of the latent hypnozoite stage requires the use of primaquine which is a challenge to administer due to the long treatment course (usually 14 days). Also, primaquine can trigger life-threatening hemolysis in individuals with glucose-6-phosphate dehydrogenase (G6PD)-deficiency, [14] an X-linked enzymatic condition that is common in malaria-endemic settings, and in settings such as Thailand, is estimated to affect 13–17% of the population [15]. The recent availability of point-of-care G6PD testing [16, 17] has made it possible to conduct testing in the community prior to primaquine administration. However, the need to conduct blood testing carries with it operational challenges, including implementation through the use of village health volunteers (VHVs) [18]. Reluctance of community members to partake in blood testing, and inherent performance issues of any diagnostic test poses additional challenges with regards to logistics and acceptability. Also, targeting forest-fringe working populations and forest-goers (or individuals living in hard-to-reach areas) can be a challenge for testing, treatment, and follow-up [19].

This qualitative study aimed to assess the acceptability and feasibility of RDA, as implemented in the trial setting, among key public health staff, VHVs, and community members in two provinces in Thailand, and to identify the necessary improvements in RDA activities and logistical considerations required for its scale-up for successful implementation of RDA into the routine malaria program.

## Methods

### Study design

A qualitative study was performed as part of a two-arm cluster randomized controlled trial (RCT) to evaluate the effectiveness of RDA, targeting high-risk villages and forest workers, compared to standard RACD for reducing subdistrict incidence and prevalence of *P. falciparum* and *P. vivax* malaria in Thailand [8].

### Study setting

Thailand is a low malaria transmission setting with a well-developed surveillance and response system based on detailed mapping of cases to the village foci level and stratification of intervention response [20]. In fiscal year (FY) 2019, 5,859 cases of malaria were reported with 83% *P. vivax*, 13% *P. falciparum*, and 4% identified as other (including mixed species, *P. knowlesi*, and unknown); nine deaths were reported [21]. This represents a 24% decrease in total cases from FY 2018. Malaria annual parasite incidence (API) stayed largely the same in FY 2018 and FY 2019 at 8.7 and 8.6 per 1,000 total population at risk, respectively. A total of 699 ‘A1’ villages (A1 village refers to local transmission is ongoing and mosquito vectors were identified) were recorded in 42 provinces.

### Study context

**Fig. 1:**
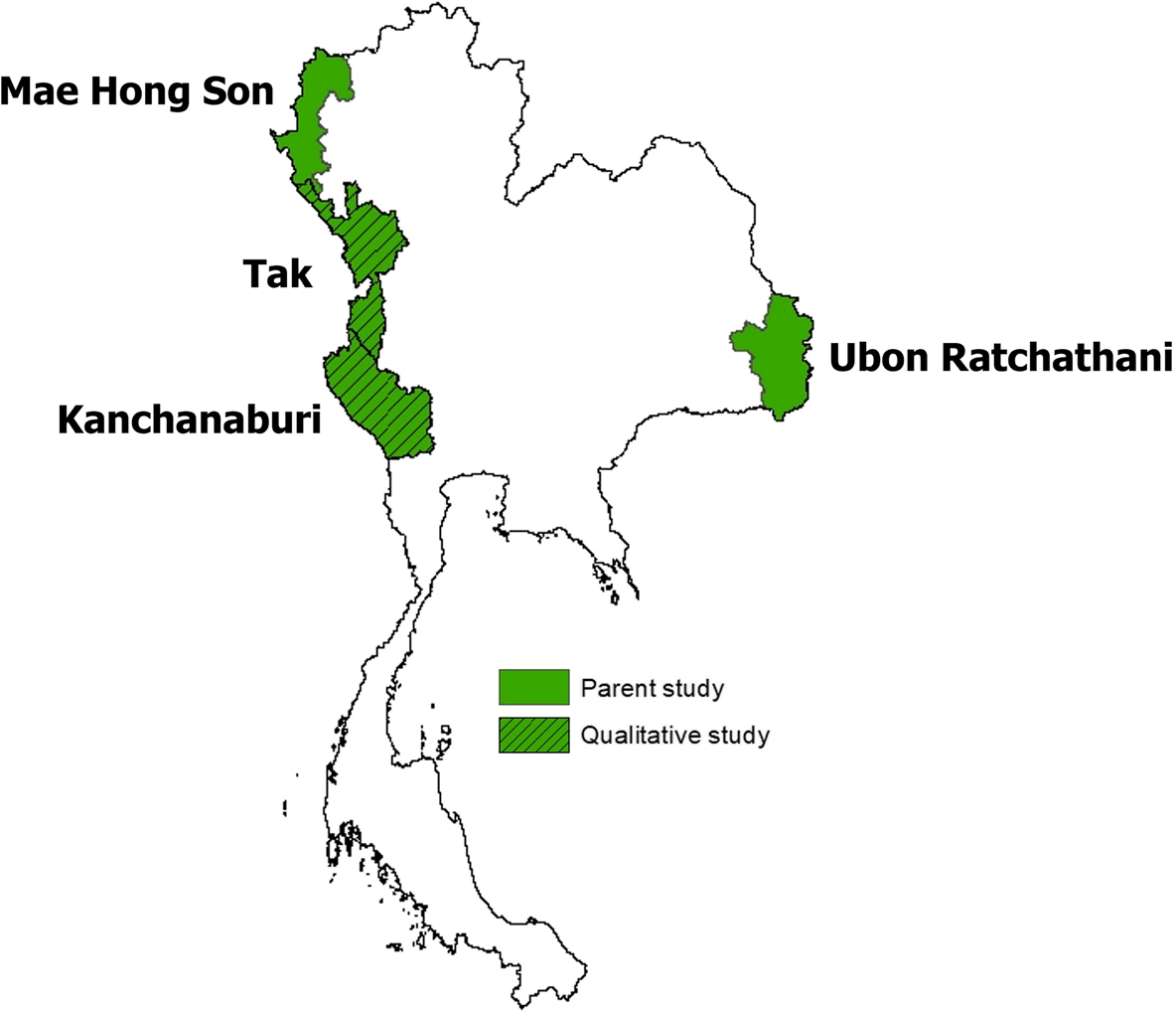
Study areas in Thailand

In the RCT, subdistricts in four of the remaining provinces with active foci in Thailand were included (Kanchanaburi, Mae Hong Son, Tak, Ubon Ratchathani), primarily bordering Myanmar to the west, and bordering Cambodia and Laos in the East. Subdistricts with at least 3 malaria cases between October 2018 and September 2019 were eligible for inclusion and stratified based upon API (high/low), total population (high/low), and geography (west/east). (Fig. 1) The RCT was carried out between November 2020—November 2021. The qualitative study was conducted from December 2021 – February 2022 in the intervention subdistricts.

**Fig. 2:**
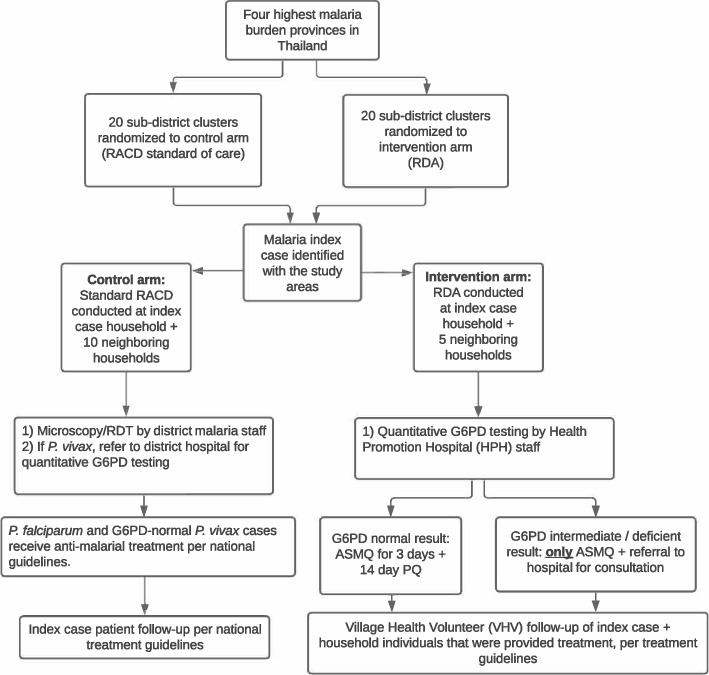
Schematic describing activities in the control and intervention arms

The control arm sub-districts received standard RACD according to national malaria surveillance guidelines [22], which follow the ‘1-3-7’ strategy for index patient diagnosis notification (within 1 day) to the online malaria information system, index patient case investigation and classification (within 3 days), and a focus investigation and tailored response (within 7 days) based on the case investigation findings and area stratification (e.g., A1 village) [20] (Fig. 2). Standard RACD was conducted by Vector Borne Disease Unit (VBDU) staff who are part of the vertical malaria program and trained in RACD procedures. Study participants were tested by VBDU staff for malaria using RDT and/or microscopy. The target screening population for the control arm was all index patient household and neighboring household members in the nearest ten households from the index patient residence up to 50 individuals within a 1 km radius.

In the intervention arm, RDA was conducted by a public health officer from the nearest health promotion hospital (HPH) (per local public health laws) with support from village health volunteers (VHVs). VHVs are part-time staff paid (1,000 Thai Baht per) to conduct healthcare-related duties in and around the villages where they live, such as hypertension monitoring, malaria treatment follow-up, Covid-19 related tasks, as well as other public health and prevention needs impacting their communities. The target population for the intervention arm was all index patient household members and neighboring household members in the nearest five households from the index patient residence up to 25 individuals within a 1 km radius. All eligible study participants were offered artesunate-mefloquine (AS-MQ) following guidance by the national medicine committee [23]. Study participants were then tested for G6PD function by taking a small blood sample and using a quantitative handheld testing device (SD Biosensor). If G6PD-normal (males ≥ 4 IU/g hemoglobin (Hb), females ≥ 6 IU/g Hb), HPH staff provided a 14-day course of primaquine (PQ) per national drug policy. For patient safety purposes, individuals testing G6PD-intermediate or -deficient were provided AS-MQ only. The first dose of AS-MQ and PQ was directly observed. Study participants were provided an envelope with the medicines, contact information for the HPH staff and VHV, a paper leaflet in local language with a list of the drug side effects and medicine card with a graphic of each medicine type to ensure accuracy of the medicine being taken according to their weight and drug regimen. To promote adherence, study participants were also given information about RDA, the potential side effects, and how to minimize them during in-person visits by health staff (on days 1, 3, 7, and 14) and as well as through village leaders. For all subsequent doses, participants or parents/guardians were provided all malaria medicines to complete the course with instructions on how to self-administer. VHVs followed up in-person all study participants who received medicine on days 3, 7, and 14 to assess adherence and any adverse events. Medicine adherence was self-reported using a structured questionnaire for individuals in each household that would be checked during the VHV follow-up visits. Adverse events among study participants were reported to the VHVs at any time during the treatment course either by text message and/or during the subsequent in-person follow-up visits.

### Study population and sampling

Purposive sampling was used to target participants included in this qualitative study and were individuals in the two provinces with the highest number of RDA events conducted during the RCT period (Tak and Kanchanaburi). Study participants who were key malaria and health staff or individuals who received the intervention during the RCT were selected for focus group discussions (FGDs) and key informant interviews (KIIs) to identify a maximum variation in responses. FGDs were conducted among HPH staff and VHVs with a target of six individuals per FGD. HPH staff received training on conducting active case detection, and protocol specific procedures on G6PD testing and interpretation, and medicine administration, safety, and adherence. HPH staff were also in charge of administering the malaria medicines to study participants according to the national malaria treatment guidelines [23], and in the supervision by the VHVs for case management of study participants receiving medicines. VHVs played a supportive role for the HPH staff to implement the study including: 1) field coordination for response to index cases; 2) preparation of documents related to data collection; 3) reviewing the consent forms and collecting consent from study participants; 4) supporting HPH staff with the blood draw and medicine allocation; 5) conducting in-person follow-up visits of all study participants receiving medicine, and if necessary, reporting any adverse event and referral to the nearest health facility.

KIIs were conducted among malaria and health staff at the provincial and district health offices. These staff are responsible for malaria at the provincial and district levels and have a key role as a supervisor and coordinator to ensure malaria activities (routine and particularly for this study) were conducted per protocol. Communities that received RDA during the RCT were targeted for KIIs and included individuals who had received the intervention and reported visiting the forest or forest-fringe for occupational purposes (foraging, gardening crops, hunting or rubber tapping) and may have stayed overnight. Participants were eligible for study inclusion if they were over 18 years old, participated in either receiving or implementing the RDA activities, and provided verbal informed consent before the interview, including permission to audio record the interviews.

### Data collection

Interview guides for FGDs and KIIs were adapted from previous RDA trials, [12, 13] and modified based on this study context and reviewed by in-country study and program staff. Guides were developed in English and translated into Thai then reviewed and confirmed by study and program staff to confirm that the translations were accurate. Interview guides were also pre-tested among researchers with qualitative experience prior to data collection. Interview guides (Additional file 1) were tailored to each respondent group which explored: 1) their roles and responsibilities in RDA; 2) perceptions of G6PD testing and referral; 3) drug adherence and safety; 4) acceptability of and attitudes toward RDA; 5) feasibility and barriers to implementing RDA; and 6) impacts due to Covid-19. FGDs and KIIs were conducted remotely via *Zoom* and by phone due to Covid-19 pandemic restrictions. Once informed consent was obtained, semi-structured interviews were conducted by the Thai social scientist research team (KS, TP, NB) in local Thai language. All team members were trained on the RCT study activities to facilitate additional probing and follow-up during the interviews. Interviews were no more than one hour and were audio recorded to review anything that was discussed.

### Data analysis

KIIs and FGDs were led by experienced facilitators supported by a trained notetaker. Each interview facilitator was accompanied by a notetaker who took notes, recorded audio, and observed the KIIs and FGDs. All facilitators and notetakers were instructed by and introduced to the study protocol, data collection methodologies, and ethical considerations for protecting the confidentiality of study participants. Notetakers were advised to observe body language, the presence of other individuals during the interviews, and the surrounding environment. Audio recordings of the FGDs and KIIs were transcribed into Thai transcriptions, then translated into English. The transcriptions were reviewed, coded by two separate researchers, and gathered into key themes per the study objectives manually. If any questions arose the audio recordings were re-visited to confirm what was said. A draft codebook was developed with both a priori codes from the interview guides and themes that emerged from reviews of the summaries and notes. Thematic analyses were conducted on notes of participants’ responses and themes determined. Content was analyzed to identify themes by exploring, interpreting, and categorizing the data. Information from different primary (interviews) and secondary (provincial malaria data and reports) sources was compared to identify consistency in their responses. In addition, Dedoose qualitative data analysis software (version 9.0.62) was used to perform content analysis to obtain findings and recommendations [24]. Analysis was led by the design of the interview guides and were as follows: 1) perceptions and knowledge of G6PD testing; 2) drug regimen adherence and tolerability; 3) acceptability of and attitudes towards RDA; 4) feasibility of implementing RDA; and 5) obstacles to RDA implementation.

## Results

A total of 13 KIIs and 8 FGDs were completed with 61 participants (13 KII and 48 FGD) from Tak and Kanchanaburi Provinces. (Table 1) Each FGD included 6 participants. The mean age of the participants was 42 years old (range of 25–59) and all participants were over 18 years of age.

**Table 1:** Characteristics of study participants

Interview method	Participant type	Tak Province		Kanchanaburi Province		Total
**Key informant interviews**		Male	Female	Male	Female	
	Province and district malaria staff	0	1	1	0	2
	Provincial supervisor	0	1	1	0	2
	Subdistrict Administrative Organization	1	0	0	0	1
	Household member	3	1	0	4	8
	Sub-total	4	3	2	4	13
	**TOTAL**	**7**		**6**		**13**
**Focus group discussions**		Male	Female	Male	Female	
	HPH staff – group 1	4	2	4	2	12
	HPH staff – group 2	4	2	4	2	12
	VHV – group 1	1	5	1	5	12
	VHV – group 2	1	5	2	4	12
	Sub-total	10	14	11	13	48
	**TOTAL**	**24**		**24**		**48**

*HPH* Health promotion hospital, *VHV* Village health volunteer

### Perceptions and knowledge of G6PD testing

Both the HPH staff and VHVs understood G6PD-deficiency, the need for G6PD testing prior to treatment, as well as the importance of rigorous follow-up of study participants to monitor potential adverse events. Previous experience with malaria infection and treatment were highlighted as a reason for their participation in this study. A VHV worker noted,

*“From experience, [community members] are afraid of malaria and their house is near the mountain so they consent for testing.”* (FGD_VHV_K_07)

Household members who participated in the RCT study received information on G6PD-deficiency and testing by the HPH staff. However, most study participants did not have the knowledge that G6PD testing was necessary before taking any malaria medicines, particularly PQ. A VHV noted that prior to the study when G6PD testing was not widely available,

*“The village may not know about ‘G6PD’, so I will ask them whether they have any Coke-color urine or other dark urine while taking primaquine. If yes, I suggest to them to go to the hospital.”* (FGD_VHV_K_07)

VHVs had knowledge that individuals with G6PD-deficiency should refrain from consuming certain foods, particularly legumes.

*“Checking [on] G6PD….the doctor said whether to eat or not eat pea for the one with deficiency. If the result shows deficiency, we cannot eat pea or legumes. [There will be] broken red blood cells.”* (FGD_VHV_K_07)

One HPH staff noted,

*“There were a lot of cases of G6PD deficiency. First, each participant’s blood was tested for G6PD deficiency. The majority of participants cooperated well, but [later] some experienced side effects and did not continue taking the medications.”* (FGD_HPH_T_01)

### Drug regimen adherence and tolerability

A majority of study participants completed the full treatment regimen of AS-MQ (86%, *n =* 558) and PQ (67%, *n =* 258). Reported side effects of the malaria medicines included dizziness, nausea, headache, vomiting, poor appetite, and weakness. No serious adverse events were reported during the parent RCT (personal communication Jintana Chaiwan). Study participants who experienced a side effect during RDA tended to stop taking the medicines causing incomplete and discontinued medicine regimens. A VHV interviewed noted,

*“The [participants] cooperated but when they took medicine and had allergy, they would stop [taking the medicine].” (FGD_VHV_K_07)*

One-third of those who received PQ did not complete the full treatment course. One household member interviewed noted that side effects started early when both AS-MQ and PQ were being administered.

*“There were symptoms after taking the medication for only 2 days, and we could not continue because we had side effects, which were muscle weakness, hand tremors, fatigue, fainting, and the inability to stand, sit or lie down. When I stopped taking the medication, my symptoms improved.”* (KII_COM_K_09)

A HPH participant interviewed noted,

*“Medicine was taken but was not completed because of dizziness and nausea. So, they did not continue the medicine.”* (FGD_HPH_K_05)

A VHV commented,

*“Some people feel dizzy when taking the medicine. Some people experience side effects, including nausea and vomiting, causing them to stop working that day.”* (FGD_VHV_K_07)

A household member commented,

*“Some people have side effects from taking the medication. Some of them have no side effects. It depends on each person.”* (KII_COM_T_04)

A HPH staff person interviewed noted that,

*“A negative view from the community was only with the people taking medicine who have side effects. The side effects made them unable to work so they did not want to join the project.”* (FGD_HPH_K_06)

Related to the malaria medicine follow up, a VHV noted,

*“[The] doctor gave the medicine and the VHV followed to check whether the participant finished all the medicines or not. We also checked whether [the participants] took the medicine correctly. Any dizziness or vomiting? Then we would provide suggestions.”* (FGD_VHV_T_03)

### Acceptability of and attitudes towards RDA

RDA was well accepted by the participants interviewed in this study. Most household members who were interviewed accepted having the index patient household members and five neighboring households take malaria medicines without prior malaria testing. Of those invited to participate, 98% agreed to participate (unpublished) in G6PD testing, a requirement to participate in the RDA study. A household member explained,

*“If [the household member] accepts and understands, this will be a protection to not get infected or have more [malaria] patients.”* (KII_COM_T_13)

VHVs, who live among the communities they serve conducting healthcare-related tasks, noted,

*“[We received] good feedback. Previously of what I have found [is that] only malaria cases have treatment…just give treatment to malaria infected case. But for this [study], the question was about why people came to take good care of them? Only one get malaria but many came to take care [and provide treatment to more people]. All people from five houses said the same.”* (FGD_VHV_T_04)

Another household member interviewed noted,

*“No one refuse, [and] provide cooperation.”* (KII_COM_K_12)

Public health staff from the Provincial Health Office provided a similar sentiment of the attitude towards RDA among the targeted population,

*“I feel that [the target population] were happy as people come to take care of them. They gave good cooperation both from Thais and migrants. Villagers in the community never refused, they cooperated well.”* (KII_PHO_K_07)

Attitudes toward RDA among VHVs was generally positive.

*“[It] is good to have a project like this. There is patient follow up so they will take medicine and give them health education. Malaria cases will decrease.”* (FGD_VHV_T_03)

One VHV described the RDA study activities as self-protection for household members:

*“I suggested taking this medicine as protection. If there is a malaria infection, the medicine will kill [the infection] and not transmit to others….and if [household members] have malaria, they cannot go to work. If the children have malaria, parents must stay with them in the hospital and waste their money.”* (FGD_VHV_T_04)

Another VHV noted that access to healthcare, especially for malaria treatment, among ethnic groups near the border and in hard-to-reach areas was an issue that encouraged their participation in this study because treatment was provided:

*“In the Bong Ti area, they were very happy. One reason is their ethnic group could not get access to malaria treatment, so they cooperated well for all [study activities].”* (FGD_VHV_K_07)

A malaria clinic person (VBDU staff) noted,

*“People in the remote mountain [areas are] afraid of [malaria] so they take medicine.”* (KII_VBD_T_02).

However, some participants had questions about the RDA activities and the individuals targeted:

*“Staying near the patient’s house, how many houses, how many people that need to take medicine? Five houses near the patient’s house even though no illness or negative for infection but they must take medicine?”* (FGD_VHV_K_07)

Furthermore, some household members refused to take the malaria medicines as they were scared and had the belief that malaria medicine is not needed because medicine is only for sick people. Many of these refusers were not in a high-risk group (e.g., forest goers) or have never had malaria and stay near the household residence. Taking medicines to treat a malaria patient is normal. However, some neighbors who have not taken malaria medicine before were afraid of the medicine’s side effects when the staff visited for blood draw and to provide medicine during RDA. One VHV noted:

*“The [household members] consented to take medicine when we explained. Only one time a participant said not to take medicine. The person stated they were scared.“* (FGD_VHV_T_03)

*“They asked why they have to take medicine since they do not have malaria? It seems they were educated on this but [did not want] to take medicine and then have dizziness?”* (FGD_HPH_K_05)

Some household members with underlying health conditions were afraid to take the malaria medicines without a known malaria infection as they were concerned about severe side effects.

*“People with underlying diseases are scared of taking medicine.”* (KII_VBD_T_02).

*“I think there is a risk among elderlies. If we let them take medicine such as my mom who is 45 years old, this normal healthy person [could turn] to a weak person.”* (KII_COM_K_09)

*“From all cases, only one did not take the medicine. He feared to [take the medicine] as he has hypertension. He [is] afraid of side effects of chloroquine and primaquine which are nausea, dizziness, and palpitation in some cases.”* (FGD_VHV_T_03)

### Feasibility of implementing RDA

HPH staff interviewed from the two study provinces reported that VHVs have the potential to perform G6PD testing and referral to the district hospital as needed but require more trainings and education in addition to supervision by the HPH staff. Currently, VHVs were not confident in their abilities to implement RDA activities safely without additional training and supervision from HPH staff since RDA would be a new activity for them. One VHV noted,

*“Now we’re still not confident [in conducting RDA] because we haven’t practiced it yet. However, VHVs will be able to do it, but we think there must be an HPH officer as well because it will make the villagers feel more confident in receiving the service. There should also be training for the VHVs on blood drawing and dispensing medicines.”* (FGD_VHV_K07)

One VHV noted:

*“In the future, training must be done. Training and practice and let the VHV do the activity by themselves with the supervision of a doctor [is ok].”* (FGD_VHV_K_07)

Most of the household participants accepted the study activities for G6PD testing and allocating the malaria medicines; however, some interviewed noted suspicions of strangers and individuals not from their communities. A VHV commented on the need to have the trust of the villages in providing RDA as a public health service, since VHV staff have not historically been able to draw blood or dispense medicines.

*“I would like the HPH staff to join us because we will gain confidence from the villagers first, and if the villagers are confident [in our ability for this new activity], we can do it ourselves.”* (FGD_VHV_T02)

Some household members raised concerns about participating in RDA but without having illness.

*“Villagers in the community said why do they have to take medicine when they do not have malaria?”* (KII_COM_T_06)

HPH staff and VHVs suggested that by having VHVs, who also live within the communities they serve, as implementers of a malaria activity like RDA, fellow community members may be more motivated and receptive to the RDA activities being implemented.

A Subdistrict Administration Organization executive in a KII reported that the role of the Local Administration Organization (LAO) is assigned with the prevention and control of communicable diseases. The LAO can support the budget and relevant organizations for implementation of RDA including materials, equipment, health supplies, etc. according to the local needs and would be able to drive the promotion of community member’s knowledge and understanding about RDA.

### Obstacles to RDA implementation

#### Logistical challenges

Challenges for follow-up by VHVs were noted in the interviews for the intervention arm due to the number of households that are required for follow-up at days 3, 7, and 14 for PQ (index case and surrounding five households per protocol, approximately 25 individuals) as well as many documents for record keeping. In the control arm, follow-up is conducted only with the index case patient based on their treatment diagnosis. Both control and intervention arms experienced logistical difficulties related to treatment follow-up including difficulty by the VHVs related to travel to the villages and communications about the study procedures. Remote areas had issues with a consistent phone signal making follow-up even harder.

*“The obstacle that we faced is no phone signal, so the communication and the travel are quite hard. The roads are bad.”* (FGD_HPH_T_01)

*“The problem and obstacle is about no internet in some areas. No electricity.”* (KII_PHO_K_07)

Some VHVs noted that they had to wait and visit community members in the evening after they returned home from work to ensure the highest coverage of the target population. And that the follow-up is even more difficult late at night in areas with no electricity or lights so the VHVs needed candles and their own lights to follow-up at each household.

*“If the patients go out [for] work, we wait until they come back after work.”* (FGD_VHV_T_04).

Travel difficulties to the harder to reach areas may include ranger camps and remote villages including dirt roads that go deep into the mountains. Participants noted that these areas, although difficult to reach, can usually be accessed by motorbike. However, during the rainy seasons, VHVs must walk to reach individuals in the forest, making follow-up in these areas a challenge.

*“It is quite troublesome, a bit harder in the raining season. Slippery road during travel in rainy season.”* (FGD_VHV_T_04)

*“Another trouble is travel problem. We had to ride a motorbike for 20 kilometers on a dirt road for follow up the taking of medicine. Normally [we] can access by car but need to walk 2 days during rainy season.”* (FGD_VHV_K_07)

### G6PD testing

During the RCT, HPH staff performed the G6PD testing and medicine dispensation per the national treatment guidelines with support from the VHVs. VHV staff highlighted that they were unsure whether they will be assigned G6PD-related duties in the future, including testing, medicine dispensation, and follow-up. VHVs noted their hesitancy with G6PD testing because they do not want to mis-read the results and were not yet confident in their skills for this activity. Furthermore, one VHV noted that there is apprehension among the community for VHVs to carry out G6PD testing,

*“The difficulty is that villagers do not trust [VHVs to conduct G6PD testing].”* (FGD VHV_K_07).

Participants confirmed that the G6PD testing should be done, but preferred to have HPH or VBDU staff accompany them to gain the villager’s trust in the VHV’s ability to carry out the activities since VHV staff have not historically conducted G6PD testing.

### Program resources

Limited budget resources in Tak and Kanchanaburi Provinces were reported by health staff interviewed, including the lack of equipment and diagnostic tests required. Currently, this equipment is used only at the hospital and malaria clinic (VBDU).

*“Currently, G6PD test devices are at district hospital only and not at HPH or broken or cannot [be] used.”* (KII_PHO_T_01)

The budgets for training and knowledge awareness for the RACD and RDA activities was noted to be limited. Additionally, insufficient travel budget for the hard-to-reach areas for public health officers, and HPH staff hampered responses to malaria index patients due to other public health priorities (e.g., Covid-19). Participants noted that if budget exists for these activities, implementation will be more effective.

### Personnel and training

The number of malaria-specific personnel in Thailand is gradually decreasing as the malaria burden also reduces. At the same time, malaria activities are being decentralized from a longstanding vertical program (VBDUs) and the role of malaria diagnosis and treatment is being transferred to the HPH staff, as well as the VHV staff who play a supportive role in case management follow-up. Participants interviewed noted a lack of VHV staff overall and their experience in conducting malaria control activities. Furthermore, it was noted that many VHV staff are retiring, and some have limited understanding of how to use data collection technology such as hand-held tablets. One HPH staff noted,

*“[An] obstacle on human resources is [having] new VHVs. The newcomers may not be familiar with malaria and never had experience about malaria.”* (FGD_HPH_T_02)

The Covid-19 pandemic has also exacerbated the issue of a lack of qualified personnel working on malaria as many of the public health officers who are trained on malaria were required to focus their efforts on the pandemic response.

*“There was no malaria field work during COVID-19. We were inconvenienced to [respond to a malaria index case] as we also need staff to be at the HPH. Not enough [HPH staff] to go out [to the field for follow up].”* (FGD_HPH_K_05)

Participants highlighted the need for VHV training, particularly those who are new to malaria. Suggestions were made to provide education to VHVs and conduct field practice sessions to understand the fundamental aspects of malaria, blood draws to test for G6PD deficiency, and to provide medicines to the higher risk groups and household members around an index patient safely.

*“Yes, [the VHVs] should pass a training if they did not have any training [yet]. If this is [their] job, training from [HPH] staff to VHV [is needed]. Some VHVs were able to do but some could not. We have selected VHVs with potential and [who are] able to learn and implement.”* (KII_VBD_K_08)

One HPH staff noted,

*If the VHV comes to work in this role, it would be good to ease the burden of HPH staff. But, VHV is not specialized in blood drawing and drug administration, so they need HPH staff to support them.”* (FGD_HPH_T_01)

## Discussion

RDA has the potential to minimize limitations in diagnostic sensitivity, particularly in near elimination settings like Thailand. This qualitative study found that RDA targeting within and around the household and forest-going travellers was generally accepted among the individuals interviewed in border and forest-fringe areas that participated and the HPH staff and VHVs who implemented RDA. Participation in RDA was largely driven by: 1) a fear of contracting malaria; 2) the individual- and community-level prophylactic protection of the malaria medicines; and 3) the increased access to health care from HPH and VHV staff. Implementing RDA was noted to be largely feasible; however, more training for the VHVs, additional malaria personnel and resources, and a transfer of roles for malaria activities from the HPH to the VHV staff to optimize public health operations were perceived important by the study participants interviewed. Study participants highlighted the importance of monitoring the side effects of the malaria medicines for their safety, particularly if underlying health conditions exist. Study participants also noted the need for more community and healthcare provider education (particularly for VHVs) and sensitization related to the purpose of RDA due to concerns raised about being asked to take malaria medicines without having illness.

The effectiveness of an intervention such as RDA primarily depends on target population coverage, strong community engagement, and adherence to the full treatment course [9]. Community motivation to participate in RDA is crucial to attain higher levels of coverage. In areas of declining malaria transmission, where infections tend to be low-density [25, 26] and asymptomatic [27, 28], the low perceived threat of malaria may outweigh risk of taking medicines presumptively. Most study participants were motivated to participate because they believed the malaria medicines would protect them and their community from contracting malaria. Others remarked that they were familiar with malaria patients receiving treatment, and were appreciative that the household members residing around the patient were also receiving medicines to prevent them from getting malaria, as were those living near the mountains and forested areas, which may put their community at a higher risk of infection [29]. Access to health care such as malaria medicines, particularly for minority ethnic groups living in remote and forested areas who may have an increased risk of exposure to malaria, [30] was highlighted as an important motivator to participate in RDA.

Some participants questioned why they were asked to take medicines without signs of malaria illness, while others noted underlying conditions such as hypertension and were worried about contraindications with the medicines. Taking prophylaxis is common practice for immune naïve individuals traveling to areas with known malaria transmission, [31] but not typical for those living in settings that have ongoing or recent history of malaria transmission. HPH staff and VHVs provided education on the purpose of taking the medicines, including the possibility of asymptomatic infections that may exist, [32, 33] but more community and healthcare provider education and sensitization (e.g., outreach, community meetings, etc.) on the purpose of RDA will be critical to its future success. Importantly, understanding the target populations and providing appropriate information in tailored ways may support the uptake of RDA [11]. Of those who refused RDA (9/817; 1.1% unpublished), some noted they did not want to have their blood drawn or take medicine, or have not visited the forest and typically stay near the household residence or not had malaria. Clustering of *P. vivax* infections around the household has been shown in some *P. vivax* dominant settings, [34]; however, evidence of infection clustering has not been shown elsewhere [27, 35]. Other factors related to occupational or behavioral exposure to malaria have been shown to play a role in malaria transmission, particularly in forested areas with highly mobile populations, [36, 37] and may be better targeted for RDA activities where the risk of having a sub-clinical or sub-microscopic infection is higher [30]. Evidence from Cambodia has shown that it may be acceptable and feasible to provide malaria prophylaxis to forest goers and may be an important way to reduce persistent reservoirs of infection among the highest risk groups.[38].

In *P. vivax* dominant settings such as these study areas, where a large proportion of infections are likely to be a relapse from a previous infection [4], administering an 8-aminoquinoline regimen for radical cure of liver hypnozoites is critical. However, risk of hemolysis in G6PD-deficienct individuals is evident, [39] and is why all RDA targeted individuals should be tested to determine their G6PD function using a quantitative G6PD testing device prior to being provided any medicines. Field studies have reported around 90% diagnostic accuracy in identifying intermediate and deficient individuals and could be a reliable tool for effective radical cure of *P. vivax* malaria [17]. Participants highlighted the need to have working G6PD biosensors in closer proximity to the community for more effective implementation.

To alleviate safety concerns and increase participant safety, a rigorous pharmacovigilance program will be important. A patient-centered approach focusing on the management of those participating in RDA and any complications or side effects from the medicines to improve the safety of those individuals is critical [40]. In eSwatini, a pharmacovigilance tool was developed to support the rollout of single low dose PQ administration for *P. falciparum*, and was found to be a feasible strategy to promote safe medicine administration [41]. Due the 14-day medicine regimen with PQ, additional consideration will be needed on the frequency of follow-up. This study conducted follow-up at days 3, 7, and 14 after the initial visit on day 1, following national guidelines for the follow-up of patients receiving malaria treatment. Furthermore, when malaria medicine consumption is directly observed evidence has shown an increase in the effectiveness of PQ administration [42, 43]. Evaluating different follow-up frequencies or strategies for RDA implementation may lead to increased acceptability of and adherence to RDA and would be important future research.

Mobile health (mHealth) platforms are increasingly providing evidence that they can improve treatment adherence and support health workers [44]. In this study, and due to local Thai law, HPH staff were required to perform the quantitative G6PD testing and medicine dispensation with support from VHVs via medication adherence follow-up visits. Interviews noted that VHVs expressed hesitancy in interpreting the G6PD test results because they have not historically conducted malaria-related tasks, particularly blood drawing and medicine dispensation. Therefore, additional trainings and close supervision by HPH staff, including streamlining of information collected (possibly through a tablet app), would be important for any scale-up of G6PD testing and RDA. Transferring malaria-specific roles from HPH staff to VHVs, providing sufficient training and on-the-ground practice and being properly resourced to integrate malaria activities into community-based activities led by VHVs may help to alleviate some of the public health duties that HPH staff are required to manage (e.g., Covid-19 pandemic follow-up, vaccination campaigns, management of other diseases and conditions, responding to outbreaks). Providing full-time employment and accompanying payment for VHV staff would allow for consistent interface with community members to provide access to malaria and other health-related services, potentially improving the uptake of interventions [45]. This may also help build trust between the VHVs and those individuals receiving RDA because official public health staff from the district level have typically conducted malaria-related activities (e.g., VBDU staff). Furthermore, the proximity of healthcare providers such as VHVs that are close to remaining malaria endemic villages, particularly villages near the forest and forest-fringe and hard-to-reach areas, may support improved access to healthcare among forest-goers [46]. These hard-to-reach and forest-going populations who have greater exposure to potentially-infective mosquitoes may also be less likely to seek care. Village leaders in areas still facing the threat of malaria transmission are critical to engage with, and should have the knowledge and understanding of RDA, including the active participation of the targeted individuals, to promote a trust and acceptance of RDA [47]. Also, including known and trusted community members when administering RDA, like VHVs who live within these communities, may provide credibility and greater engagement from the community [48].

Side effects experienced from taking the malaria medicines plays an important role in the acceptability of a drug-based intervention. In this study, one-third of participants did not complete the full course of malaria medicines due to side effects, which included dizziness, nausea, vomiting, and weakness, similar to side effects reported from taking anti-malarial medications [49]. Typically, when side effects were experienced, participants reported that they stopped taking the malaria medicines. There is evidence that shortening the medicine regimen for primaquine from 14 days (0.5 mg/kg per day) to 7 days (1.0 mg/kg per day) is well tolerated in G6PD-normal individuals and may improve adherence to the medicine regimen and acceptability of RDA by the community [50]. This has important implications for a program trying to eliminate *P. vivax* relapsing infections [4]. Proper training for VHVs to provide the simple medication instructions in an understandable format as well as ways in which side effects can be mitigated (i.e., taking medicines with food) can support intervention acceptance [51]. Furthermore, engagement with village leaders on the importance of completing the medicine regimen may help support the acceptability of RDA.

There were some limitations of our study. First, recall bias could have affected the quality of the feedback provided by study participants since interviews were conducted at the end of the 12 months long RCT. We tried to mitigate recall bias by conducting the KIIs and FGDs immediately after the RCT ended. Furthermore, study participants were chosen in the two highest burden provinces where most RDA events occurred so they would have more experience conducting or receiving RDA activities. Selection bias may have been introduced by including only key public health staff and household members in study areas who agreed to participate in RDA and in the interviews. Overall, refusals to enroll in the RCT were very low (2.2%), so this may not have much impact on the findings. Additionally, the views and opinions of study participants may not completely reflect all public health staff and/or high-risk populations in the community since this study population was purposively sampled. Due to the COVID-19 pandemic and travel restrictions, interviews were conducted remotely via *Zoom* and by phone and therefore may have impacted the quality of responses compared to conducting in-person interviews. We did not evaluate the interpretation of the G6PD results by the HPH staff nor whether increased frequency of VHV follow-up would have led to increased adherence to the malaria medicines but would be important future research to possibly increase acceptability of and adherence to RDA. PQ treatment was not provided to children during the RCT because we did not have the correct tablet dose for this age group therefore restricting these findings to adults only.

Despite the limitations, the results from this qualitative study support RDA to be programmatically feasible and acceptable in the communities involved if RDA is found to be an effective strategy to reduce malaria transmission. The RCT study activities were conducted through the national malaria program, including the district health offices, HPHs, and VHVs to ensure that RDA could be replicated if it was deemed acceptable by those participating in it. Further, this study adds to a limited evidence base for the acceptability and feasibility of implementing RDA, and particularly in *P. vivax* dominant settings.

## Conclusions

A novel reactive drug-based intervention targeting household members and surrounding neighbors of a recently reported index patient in Thailand was shown to be acceptable by those receiving RDA and feasible to implement by the public health staff and VHVs included in this qualitative study. To maximize uptake of RDA, regular education and sensitization campaigns in collaboration with village leaders on the purpose and rationale of RDA will be critical. To alleviate safety concerns and increase participant safety, a rigorous pharmacovigilance monitoring program will be important. To accelerate uptake of RDA, trust between public health staff and VHVs and the communities they serve must continue to be strengthened to ensure acceptance of the intervention.

## Supplementary Information

**Additional file 1. **

## Data Availability

All relevant data are within the manuscript.

## Abbreviations

API: Annual parasite incidence
AS-MQ: Artesunate-mefloquine
FGD: Focus group discussion
FY: Fiscal year
G6PD: Glucose-6-phosphate dehydrogenase
HPH: Health promotion hospital
KII: Key informant interview
PQ: Primaquine
RACD: Reactive case detection
RDT: Rapid diagnostic test
RDA: Reactive drug administration
VBDU: Vector borne disease unit
VHV: Village health volunteer
