# Supplementary material for: Assessing the acceptability and feasibility of reactive drug administration for malaria elimination in a Plasmodium vivax predominant setting: a qualitative study in two provinces in Thailand
Source: BMC Public Health. 2023 Jul 13;23:1346. doi: 10.1186/s12889-023-15852-z (PMC10339568; doi:10.1186/s12889-023-15852-z)
Supplement: Supplementary file 1 — Additional file 1. [file 12889_2023_15852_MOESM1_ESM.zip › Additional file 1_Qualitative guides_Final/Community member interview guide_Thailand.docx]

**Form 8.15 Community Member Interview Guide**

**Assessor name: ______________________ Date: __ __ / __ __ / __ __**

d d m m y y

**Community member code: ______­­­­­­______**

**Participant occupation:___________________________**

**Interview start time: ___ ___ : ___ ___ AM / PM** (circle)

**Interview end time: ___ ___ : ___ ___ AM / PM** (circle)

**Notes: ________________________________________________________________________**

**____________________________________________________________________________**

| **Purpose:** | To explore perceptions of malaria risk and experience with malaria among community members; To assess the acceptability, and experience of reactive focal drug administration study activities among community members, including drug adherence and adverse side effects. |
| --- | --- |

**Section 1: Background and experience with malaria testing and treatment *PRIOR TO STUDY ENROLMENT***

| **NO.** | **QUESTION** | **RESPONSE** |
| --- | --- | --- |
| 1 | Have you lived in this community for at least 5 years? If not where else have you lived? |  |
| 2 | Have you had malaria in the past 5 years?  *(prior to the study enrolment)* | Yes ☐  ____ number of times  No ☐  Not sure ☐ |
| 3 | Do you think you can have malaria but not experience symptoms? |  |
| 4 | *(prior to the study enrolment)*  If you think you had malaria, were you tested? If not, why not? |  |
| 5 | *(For those that reported malaria and not tested)*  Did you take any drugs when you had suspected malaria? If yes, please list the drugs taken. |  |
| 6 | *(Regardless of testing or not)*  Tell me about your experience taking **malaria drugs**. Where did you obtain the drugs? |  |
| 7 | Do you think you or your family are at risk for malaria? Why or why not? |  |
| 8 | What characteristics or behaviors of members in your community do you think puts them at risk for malaria? *(Probe. Note: not individual names but any important social, behavioral or economic characteristics identified.)* |  |
| 9 | What do you do to protect yourself and your household from getting malaria? (*Probe*.) |  |
| **Section 2:** | **Experiences during this study** |  |
| 1 | Tell me about how you were contacted regarding this study? |  |
| 2 | Tell me about your decision to be a part of this study? What influenced your decision to participate? |  |
| 3 | Tell me what you thought about the intervention (reactive focal drug administration) when you first heard of it. |  |
| 4 | What is your understanding of why people in your community were given malaria drugs without being tested? Can you explain the reasons to me? |  |
| 5 | What do members in the community say about this malaria study and its activities?  *(Probe: positive and negative things.)* |  |
| 6 | Do you know any community members that refused to participate in this study? If so, do you know why?  *(Note: do not need individual names of people, only reasons why they did not participate)* |  |
| 7 | Is this the first time you were given malaria drugs without being tested for malaria first? Would you do it again? Why or why not? |  |
| 8 | What do you think about getting tested for malaria before you take drugs? Is it important to test for malaria first or not? Why? |  |
| 9 | Thinking back to when the study team was visiting you to conduct the intervention, do you have any suggestions on how to improve those activities? |  |
| 10 | How was your experience with the malaria drugs? How did it make you and your family feel? |  |
| 11 | What malaria treatment was provided? |  |
| 12 | Did you finish your malaria treatment? Why or why not? (*If not finished, please explain why?)* |  |
| 13 | How did you feel about the length of treatment course pills? |  |
| 14 | How did you feel about the number of pills per day? |  |
| 15 | Did you or anyone in your household have adverse/ side effects as a result of the malaria treatment?  If so, please list adverse/ side effects and describe each. |  |
| 16 | Did the study activities negatively affect you or your family in any other ways? If so, please describe how? |  |
| **Section 3:** | **Other malaria** |  |
| 1 | Would you be willing to take malaria treatment without testing for malaria before the start of the rainy/malaria season as a way to prevent malaria? Why or why not? |  |
| 2 | In what ways can those in your household and neighbors be motivated to complete their malaria treatment? |  |
| 3 | What is your overall opinion of this study? |  |
| 4 | Do you have any other suggestions for us on how we can improve our malaria work in the communities in the future to eliminate malaria? |  |
| **Section 4:** | **Covid-19 related** |  |
| 1 | What effects did the covid-19 pandemic and the government response or restrictions have on you (if any) related to visiting a malaria clinic or health promotion hospital? |  |
| 2 | Did the covid-19 pandemic impact when and how frequently you visited a malaria clinic or health promotion hospital? Please explain. |  |
| **As a reminder, all information shared with us during this interview will be kept confidential and will only be used for research purposes. No information generated from this activity will be directly associated with the individual.** | | |
