# Supplementary material for: Assessing the acceptability and feasibility of reactive drug administration for malaria elimination in a Plasmodium vivax predominant setting: a qualitative study in two provinces in Thailand
Source: BMC Public Health. 2023 Jul 13;23:1346. doi: 10.1186/s12889-023-15852-z (PMC10339568; doi:10.1186/s12889-023-15852-z)
Supplement: Supplementary file 1 — Additional file 1. [file 12889_2023_15852_MOESM1_ESM.zip › Additional file 1_Qualitative guides_Final/Provincial and Supervisor KII Interview Guide_Thailand.docx]

**Form 8.18 Key Informant Interview – Provincial and Supervisor (VBDC)**

**Purpose:** To assess the feasibility of vector borne disease center (VBDC) and vector borne disease unit (VBDU) staff to support reactive focal drug administration, including G6PD testing and drug adherence follow-up, and explore barriers to the integration of study activities in the routine malaria program.

1. Tell me about your role as provincial/district malaria staff related to this study. What are your specific job responsibilities in this role?
2. What are your thoughts on providing malaria drugs to household members and neighbors in response to an index case without testing if they have malaria?
   1. Do you see this as being helpful to the program? Why or why not?
3. Do you think the reactive focal drug administration study activities helped to decrease the risk of malaria in your area? Why or why not?
4. What do you think about the reactive focal drug administration activities conducted in this study?
   1. Were there any issues or complications you experienced? If so please explain.
   2. What changes or improvements to the focal drug administration activities should be made, if any?
5. Do you think these reactive focal drug administration activities can be conducted in a routine way? Why or why not?
   1. What additional support or trainings would be needed if village malaria workers (VMWs)s conducted reactive focal drug administration?
6. What do you think about the feasibility to conduct reactive focal drug administration among high-risk populations (HRPs) and in forest areas where they work? Please explain.
   1. What do you think would be the main barriers to conducting these types of activities among HRPs? Why?
   2. How could we overcome the barriers you mentioned?
7. What were people in the community saying about this study, if known? Please share both positive and negative comments you have heard.
8. How do you think neighbors of index case households felt about taking malaria drugs without testing first, if known? Is this acceptable to the community? Why or why not?
9. What are some ways to encourage community participation and safe implementation of reactive focal drug administration activities in the future?
10. What types of supervision do you think would be necessary to ensure reactive focal drug administration is carried out promptly and properly? Who do you think should provide this supervision? Why?
11. What kind of support or actions from leadership in your department are needed to make reactive focal drug administration a routine activity? *(Probe: for specific support/ actions from leadership.)*

**G6PD testing**

1. Do you think VMWs are able to conduct G6PD testing under HPH or VDBU staff supervision as a routine malaria case management activity? Why or why not? Please explain.
   1. What about without HPH or VDBU supervision?
2. What do you think would be the barriers to conducting routine G6PD testing by VMWs? *(Probe: financial, human resource, transport, training, other?)*
   1. How can these barriers be addressed?
3. What types of supervision do you think would be necessary to ensure G6PD testing is carried out promptly and properly? Who do you think should provide this supervision? Why?
4. What policy-level changes would need to happen to make G6PD testing a routine activity?
   1. What facilitators and barriers do you think there would be?
5. What additional resources or trainings would be needed for you or your staff related to G6PD testing, if any? How often?
6. Should VMWs be able to provide treatment with malaria drugs or refer based upon the G6PD test result? Please explain.

**Malaria case management**

1. What do you think are the biggest barriers to radical cure (14-day primaquine) for *P. vivax* infection?
2. What are some ways to improve the case management and follow-up for *P. vivax* infection?
3. What additional resources or trainings would be needed for you or your staff on malaria case management for presumptive treatment, if any? How often?
4. In what ways do you think we can overcome barriers to community members not completing their malaria drug regimen, particularly related to a reactive focal drug administration response?

**Covid-19 and other**

1. Was your routine work affected by the covid-19 pandemic and response? If so, in what ways?
   1. What effects did the covid-19 outbreak and the government response or restrictions have on your ability to conduct reactive focal drug administration?
2. Did participation by community members in the reactive focal drug administration intervention areas change with the outbreak of covid-19 after Songkran? *(If known)*
3. Do you think malaria case responses were impacted by covid-19 health policies? How so? *(Probe: testing and treatment, fever-seeking behavior)*
4. What is your overall opinion of this study?
5. Do you have any other information that you would like to share with us about this study and its activities?

**As a reminder, all information shared with us during this interview will be kept confidential and will only be used for research purposes. No information generated from this activity will be directly associated with the individual.**
