# Supplementary material for: Assessing the acceptability and feasibility of reactive drug administration for malaria elimination in a Plasmodium vivax predominant setting: a qualitative study in two provinces in Thailand
Source: BMC Public Health. 2023 Jul 13;23:1346. doi: 10.1186/s12889-023-15852-z (PMC10339568; doi:10.1186/s12889-023-15852-z)
Supplement: Supplementary file 1 — Additional file 1. [file 12889_2023_15852_MOESM1_ESM.zip › Additional file 1_Qualitative guides_Final/VMW FGD Guide_Thailand.docx]

**Form 8.14 Focus Group Discussion – Village Malaria Workers (VMWs)**

**Purpose:** To understand the feasibility and acceptability of village malaria workers to conduct reactive focal drug administration in the community and among high-risk populations, including drug adherence, G6PD testing, and adverse event experience.

**Work duties**

1. Tell me about your role as a VMW related to this study. What are your specific job responsibilities as a VMW? *(Probe.)*
2. Do you think the reactive focal drug administration study activities helped to decrease the risk of malaria in your area? Why or why not?
3. What do you think about the reactive focal drug administration activities conducted in this study?
   1. Were there any issues or complications you experienced? If so please explain.
   2. What changes or improvements to the focal drug administration activities should be made, if any?
4. Do you think the reactive focal drug administration activities can be conducted by VMWs in a routine way? Why or why not?
   1. Would you require supervision or assistance from other health staff? Why? Who?
   2. What additional support or trainings would be needed if VMWs conducted reactive focal drug administration?
5. What do you think about the feasibility to conduct reactive focal drug administration among high-risk populations (HRPs) and in forest areas where they work? Please explain.
   1. What do you think would be the main barriers to conducting these types of activities among HRPs? Why?
   2. How could we overcome the barriers you mentioned?
6. What were people in the community saying about this study? Please share both positive and negative comments you have heard.
7. How do you think neighbors of index case households felt about taking malaria medication without testing first? Is this acceptable to the community? Why or why not?
8. Were there any particular groups who did not want to participate in the study? Which groups and why do you think some people refused to participate? *(Note to interviewer: not names of specific individuals but types/groups of people.)*
9. What are some ways to encourage community participation and safe implementation of reactive focal drug administration activities in the future?

**G6PD deficiency and testing**

1. Please tell me what you know about G6PD deficiency. What risks are associated with G6PD deficiency? *(skip if not known*)
2. Do you think VMWs would be able to conduct G6PD testing under the HPH staff supervision as a routine activity? Please explain.
3. What do you think would be the main barriers to G6PD testing as a routine activity?

*(Probe: financial, human resource, transport, training, other?)*

- 1. How can these barriers be addressed?

1. Do you ever refer people to district hospitals for primaquine treatment?
2. If so, in what situations?
3. What might motivate them to go?
4. What might prevent them from going?

**Drug adherence in intervention arm**

1. Do you track all participants to determine if they completed their malaria drugs? Please describe this tracking process.
2. When malaria drugs were provided to study participants (and who were not tested for malaria) did they finish the drugs?
   1. What might have prevented them from finishing the malaria drugs?
      1. Do you consider this a problem?
   2. What might motivate them to finish the drugs?
   3. If study participants do not finish the malaria drugs, typically how many days after beginning the drugs do they stop? *(if known, can be approximate)*
3. Did you visit individuals taking the study drugs at their home? Why or why not?
4. Are there some people or situations where it might be more difficult to conduct follow-up visits? Please describe those situations. *(Probe for high-risk populations working and living in forest.)*
5. In what ways do you think we can overcome barriers to community members not completing their malaria drug regimen, particularly related to a reactive focal drug administration response?

**Adverse events**

1. Did anyone involved in the study that you know of experience any adverse events while taking malaria drugs? List as many adverse events experienced as possible.
2. Did they think the adverse event(s) was related to the G6PD testing and/or malaria drugs? Why or why not?

**Covid-19 and other**

1. Was your routine work affected by the covid-19 pandemic and response? If so, in what ways?
   1. What effects did the covid-19 outbreak and the government response or restrictions have on your role as a VMW?
2. Did participation by community members in the reactive focal drug administration intervention areas change with the outbreak of covid-19 after Songkran?
3. Do you think malaria case responses were impacted by covid-19 health policies? How so? *(Probe: testing and treatment, fever-seeking behavior)*
4. What is your overall opinion of this study?
5. Do you have any other information that you would like to share with us about this study and its activities?

**As a reminder, all information shared with us during this interview will be kept confidential and will only be used for research purposes. No information generated from this activity will be directly associated with the individual.**
